# Effect of Cr Content on Corrosion Resistance of Ni–xCr–Mo Laser-Cladding Coatings under H_2_S-Induced High-Temperature Corrosion Atmosphere

**DOI:** 10.3390/ma15051885

**Published:** 2022-03-03

**Authors:** Congcong Liu, Zongde Liu, Yuan Gao, Xinyu Wang, Chao Zheng

**Affiliations:** 1 Key Laboratory of Energy Transfer and System of Power Station of Ministry of Education, North China Electric Power University, Beijing 102206, China; jylcc5613@163.com (C.L.); wangxinyu1939@163.com (X.W.); zhengkirk@aliyun.com (C.Z.); 2 Institute of Science and Technology, China Three Gorges Corporation, Beijing 100038, China; 18810178944@163.com

**Keywords:** Ni–Cr–Mo, high-temperature corrosion, H_2_S, laser cladding

## Abstract

Ni–xCr–Mo laser-cladding coatings with varying Cr content of 10, 15, 20, 25, and 30 wt.% were fabricated using a self-assembled coaxial laser-cladding device. The H_2_S-induced high-temperature corrosion tests under reductive atmosphere were conducted at 500, 550, and 600 °C. Subsequently, the influence of Cr content on the microstructural evolution and corrosion resistance of the Ni–xCr–Mo coatings was investigated. The experimental results revealed that 30 wt.% Cr is the limited maximum content that forms the suitable morphology of coatings without large prominent pores and cracks during the fabrication process, and 15 wt.% Cr corresponds to the critical minimum content for excellent corrosion resistance, as implied from the variation tendency of the corrosion weight-gain curves. Moreover, a two-layer structure of the corrosion scales was observed in the Ni–xCr–Mo laser-cladding coatings, which was primarily caused by the selective corrosion between the Ni and S and Cr/Mo and O.

## 1. Introduction

Ni–Cr–Mo alloys such as Hastelloy C22, Hastelloy C276, C-4, and Inconel 625 have garnered considerable attention owing to their high corrosion resistance against pitting, crevice, and stress corrosion [1,2,3,4,5,6,7]. In addition, Ni–Cr–Mo alloys are widely used across the modern industry because of their excellent corrosion resistance toward chlorine, hot pollution solution, strong acid, strong alkali, organic solvents, and high-temperature corrosion, thus establishing their ability of exceptional corrosion resistance [8,9,10,11]. Furthermore, the Ni–Cr–Mo alloys can be used in the H_2_S-induced high-temperature corrosion atmosphere caused by the low-nitrogen combustion in garbage power plants or coal-fired power plants [12]. However, the high cost of Hastelloy C22 bulk limits its industrial applications.

Recently, surface engineering technology has been used to prepare Ni–Cr–Mo cladding coatings [13,14,15,16,17,18,19,20,21]. Vignolo et al. [22] fabricated a Hastelloy thin film by XeCl-pulsed laser ablation. Liu et al. [1] reported that the corrosion resistance ability of Hastelloy C22 prepared using a pulsed optical-pumped Nd:YAG laser was equal to that of the bulk one. In particular, the laser-cladding technology was used to prepare the Ni–Cr–Mo cladding coating owing to its high power, strong metallurgical bonding with matrix, small heat-affected zone, and small matrix deformation [13,14,20,21]. Wang et al. [17,23,24] studied the characteristics and corrosion resistance of laser-cladded C22 coatings, including the fabrication optimization parameters, to improve the corrosion resistance. However, the laser-cladding technology becomes a competitive preparation process for Ni–Cr–Mo coatings because it can save costs and maintain corrosion resistance as well.

More specifically, the amount of chromium and molybdenum is an essential factor contributing to the corrosion resistance of Ni–Cr–Mo alloys. Numerous studies have demonstrated that the corrosion resistance of Ni–Cr–Mo coatings can be effectively improved by increasing the Cr, Ni, and other alloying elements because the alloy elements aid in the formation of a dense protective oxide scale on the matrix surface [25,26]. However, the existing research on the corrosion resistance of Ni–Cr–Mo alloy is mainly focused on commercial alloys, and the influence of Cr on the corrosion resistance of Ni–Cr–Mo laser cladding has not been extensively studied.

Therefore, this study aims to detail the corrosion mechanisms of Ni–xCr–Mo laser-cladding coatings with varied Cr content in a simulated low-NOx combustion environment at 500–600 °C. The phase constitution and microstructure evolution of the Ni–xCr–Mo laser-cladding coatings were analyzed before and after corrosion tests. Furthermore, we identified the characteristics and discussed the influence of Cr content on the corrosion kinetic curves, corrosion products, and corrosion mechanisms. The optimistic Cr content for Ni–xCr–Mo laser-cladding coatings was acquired, which can provide excellent corrosion resistance, and the present experimental results will aid in developing a promising corrosion prevention Ni–xCr–Mo laser-cladding coating for coal-fired boilers.

## 2. Materials and Methods

Ni–xCr–Mo cladding powders (offered by Beijing General Research Institute of Mining and Metallurgy, Beijing, China) containing 10, 15, 20, 25, and 30 wt.% Cr were prepared, and the detailed chemical compositions with sample names are listed in Table 1. The Ni–xCr–Mo laser coatings were fabricated using a self-assemble coaxial laser-cladding device equipped with a 2000 W high-power fiber laser (ZKZM-2000, zKzM Laser Technology Co., Ltd., Xi’an, China), a self-designed coaxial powder-feeding nozzle that ejected three powder streams, and an argon shielding system. The schematic of the laser-cladding process is illustrated in [27]. During the fabrication of laser-cladding coatings, the 20G steel substrate with dimensions of 20 × 20 × 5 cm was polished and cleaned with acetone. Following prior research [27,28], the laser-cladding parameters were set as follows: beam spot was 1.4 mm in diameter, laser power was 2000 W, overlap ratio was 40%, laser scan speed was 8 mm/s, and the flow rate of argon was 5 L/min. In order to reduce the negative effect of dilution rate on the corrosion resistance of the laser coatings, the thickness of the laser coating prepared on the surface of the 20 G substrate by the multitrack cladding process was set at 5 mm. The specimens with a size of 20 × 10 × 3 mm of laser cladding coating were obtained from the top 3 mm of the laser cladding coatings.

Subsequently, isothermal corrosion tests were conducted in a horizontal tube furnace (SK2-6-12, FNS (Beijing) Electric Furnace co., Ltd; Beijing, China) for 168 h (24 h × 7 days) at 500, 550, and 600 °C. To simulate the reducing environment caused by low-nitrogen combustion in coal power plants, a synthetic gas mixture containing 0.2 vol.% H_2_S, 0.1 vol.% O_2_, and 99.7 vol.% N_2_ was selected as the high-temperature corrosive medium, simulating low-nitrogen combustion of coal power plants. In addition, the gas flow rate was set at 100 mL/min. Prior to the corrosion test, all samples were grounded using SiC sandpaper from 200# to 1200# and then cleaned using an ultrasonic cleaner with an acetone medium. Moreover, a mass balance with an accuracy of 0.0001 g was used to measure the mass gain after every corrosion cycle. Overall, the average values of the three specimens were considered the final mass-gain data for each sample. The mass gain (mg/cm^2^) highlighting the corrosion resistance of specimens was calculated as [28]:

(1) γ=ΔmA

where Δ*m*(g) denotes the average cumulative mass gain of three parallel specimens determined simultaneously with increasing corrosion time, and *A* represents the surface area of the samples.

The microstructure of the prepared laser-cladding coatings was examined prior to the corrosion test using bulk samples that were machined from the coatings using a linear cutting machine. Thereafter, the cross-section of each sample was ground, polished, and etched with corrosive liquid of aqua regia for 30–40 s. Before and after the corrosion tests, the microstructure of the samples was observed using a scanning electron microscope (SEM, FEI Quanta 200F, Brno, Czech Republic), equipped with an energy dispersive X-ray spectrometer (EDS, EDAX, Mahwah, NJ, USA) for chemical analysis. Additionally, phase analysis was performed on an X-ray diffractometer (XRD, Rigaku D/Max-2400, Tokyo, Japan) with Cu–Kα radiation between 2θ angles of 10° and 90° at a scanning rate of 8°/min. Furthermore, the surfaces of specimens for X-ray diffraction analysis were ground and polished.

## 3. Results

### 3.1. Microstructure and Phase of Ni–xCr–Mo Laser-Cladding Coatings

The actual element composition of the prepared laser coatings is presented in Table 2, comparing which to Table 1 reveals that the element content during the cladding process was typically stable, without loss.

**Figure 1:**
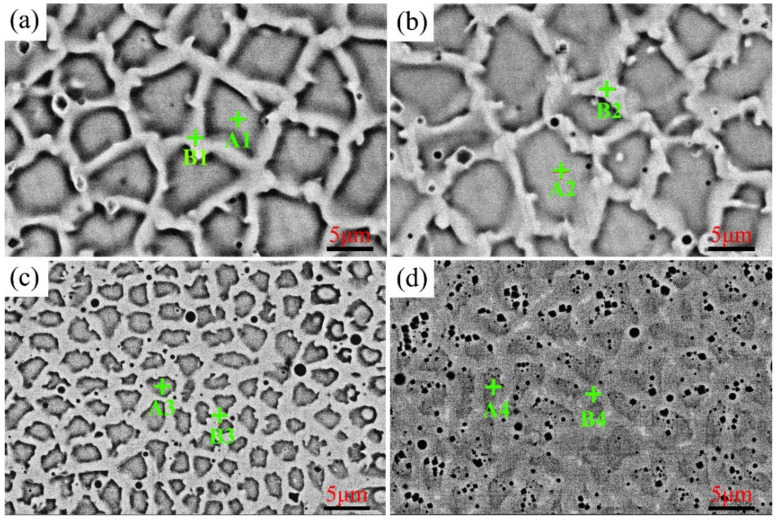
SEM micrographs of Ni–xCr–Mo coatings with varying Cr contents: (**a**) C1, (**b**) C2, (**c**) C3, and (**d**) C4.

The SEM surface micrographs of prepared Ni–xCr–Mo laser-cladding coatings containing 10, 15, 20, and 25 wt.% Cr are illustrated in Figure 1a–d, respectively. The element contents of the typical phases of the Ni–xCr–Mo laser-cladding coatings are listed in Table 3. As displayed in Figure 1, the microstructures of C1–C4 were similar because the surface is composed of an equiaxed phase (pointed out as A1) and a network phase (pointed out as B1). In particular, the width of the network phase and the number of pores in the cladding coatings increased with the Cr content. Moreover, the pores in C1, C2, and C3 were less than those in C4, and the pores in C1–C3 were primarily distributed in the network phases, whereas the pores in C4 fundamentally appeared in the equiaxed phases, as depicted in Figure 1d. The EDS results reported in Table 3 implied that the Cr and Mo contents of the network phases were greater than those of the equiaxed phase, and the Mo content of the network phase increased with the Cr content. In general, the Mo-rich network phases became coarser, and the number of pores increased as the Cr content rose from 10 to 25 wt.%.

**Figure 2:**
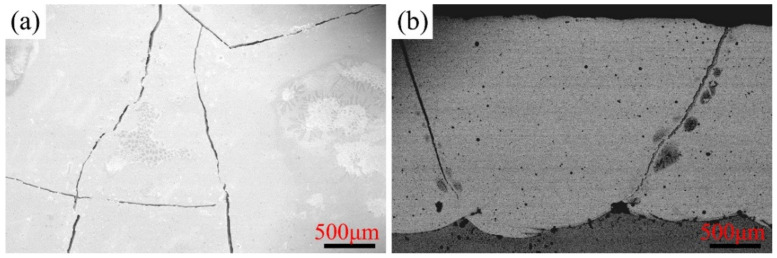
SEM morphology of C5 laser-cladding coating: (**a**) surface and (**b**) cross-section.

As portrayed in Figure 2, evident cracks appeared in the cladding layer when the Cr content increased up to 30 wt.%. The appearance of large cracks primarily resulted from the internal stress generated during the cladding process, which was caused by the excessive Cr content reducing the strength of the cladding layer. These prominent cracks yielded poor corrosion resistance; therefore, only C1–C4 are analyzed in the following sections. Consequently, the 25 wt.% Cr was considered the limited maximum content for improved morphology without large prominent pores in the Ni–xCr–Mo laser-cladding coatings.

**Figure 3:**
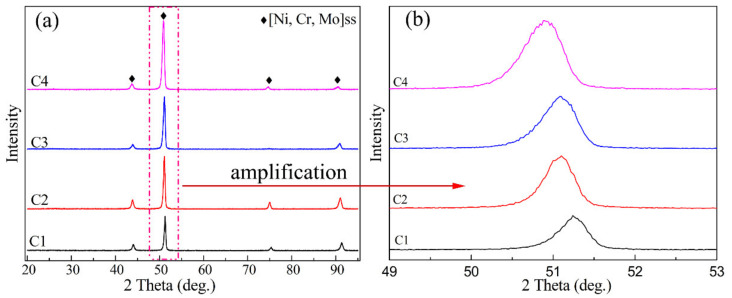
(**a**) XRD patterns and (**b**) partially enlarged patterns of C1–C4 coatings.

The XRD patterns of the prepared Ni–xCr–Mo laser-cladding coatings with varying Cr content are illustrated in Figure 3, which implied similar XRD patterns of the four laser coatings (Figure 1) and that the fundamental phase included (Ni,Cr,Mo)ss solid solution. To study the phase evolution of the Ni–xCr–Mo laser-cladding coatings, the highest major peaks of the XRD patterns for various samples were partially enlarged and presented in Figure 3b. As observed, the highest peaks shifted to the left with the addition of Cr, which could be attributed to the increase of Cr content in the (Ni,Cr,Mo)ss from 10.2 to 26.9 at.% (Table 3). According to Bragg’s Law [29], the crystal plane spacing (d) increases with the Cr content in the solid solution, thereby reducing 2θ and shifting the highest peaks of various samples to the left. Thus, the XRD patterns portrayed in Figure 3 were consistent with the EDS results of typical phases listed in Table 3.

### 3.2. High-Temperature Corrosion Kinetics Curves

**Figure 4:**
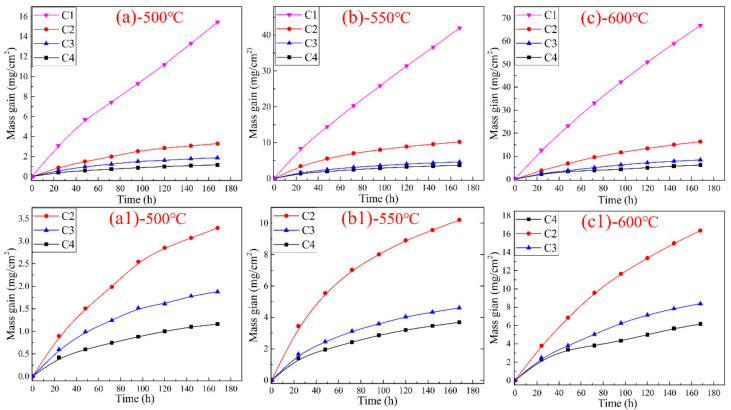
Mass gain of C1–C4 corroded in reducing atmosphere for 168 h at 500–600 °C as a function of time: (**a**) 500, (**b**) 550, (**c**) 600, (**a1**) 500, (**b1**) 550, and (**c1**) 600 °C.

The mass gain of C1, C2, C3, and C4 corroded in 0.2 vol.% H_2_S–0.1 vol.% O_2_–N_2_ for 168 h at 500, 550, and 600 °C are exemplified in Figure 4, wherein the results portrayed in Figure 4a–c indicate that the corrosion mass gain increased with the extension of time. In addition, the mass-gain curves of C1 at three distinct temperatures conformed to the linear law, which signified that the corrosion products of C1 were not adequately dense to inhibit the corrosion process during the high-temperature corrosion experiments at 500–600 °C. Thus, the corrosion products of C1 were loose and filled with pores, and the corrosive medium could conveniently pass through the corrosion products to contact and react with the matrix, resulting in a higher corrosion weight-gain rate. Moreover, the corrosion of C1 intensified with the increase in temperature. The local magnification of the corrosion weight-gain curves for C2, C3, and C4 are presented in Figure 4 (a1,b1,c1), which implied that the corrosion weight-gain curves followed near parabolic law (a detailed discussion is presented in Section 4.1) when the Cr content was between 15 and 25 wt.%. This phenomenon indicated that the corrosion products of the Ni–xCr–Mo laser-cladding coatings containing 15–25 wt.% Cr could prevent the inner matrix from high-temperature corrosion and reduce the mass gain of corrosion of the products.

Generally, at the same corrosion temperature, the corrosion resistance of the cladding layer increased with the Cr content, and the order of corrosion resistance was C4 > C3 > C2 > C1 at 500–600 °C. The influence of the Cr content on the corrosion resistance of Ni–xCr–Mo laser-cladding coatings is further discussed in Section 4.1.

### 3.3. Phase and Surface Morphology after High-Temperature Corrosion Tests

**Figure 5:**
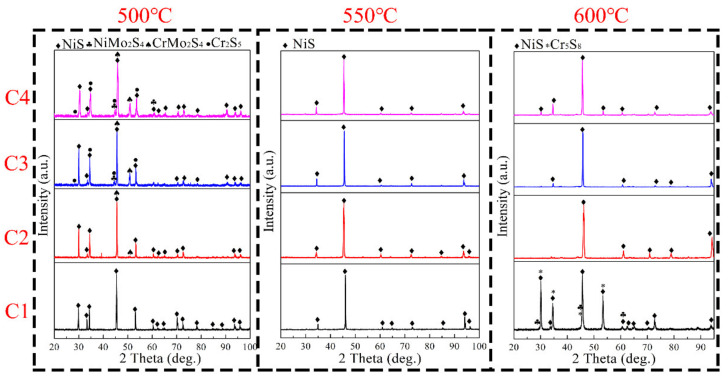
XRD patterns of corrosion products formed after high-temperature sulfur corrosion tests at 500–600 °C.

The XRD patterns for the surface corrosion products of Ni–xCr–Mo laser-cladding coatings after sulfur corrosion tests at 500–600 °C are presented in Figure 5, which imply that the major corrosion products at 500 °C are oxides and sulfides of Ni, Cr, and Mo, e.g., NiS, NiMo_2_S_4_, CrMo_2_S_4_, Cr_2_S_5_, and Cr_5_S_8_. However, the peaks for only the NiS phase were observed at 550 °C without any other corrosion products. The thick corrosion scale containing NiS on the surface inhibited the detection of corrosion products formed in the interior of the surface. In general, the specific type and content of the corrosion products of Ni–xCr–Mo laser-cladding coatings after sulfur corrosion tests at 500–600 °C could not be confirmed owing to the various metal elements present in the laser-cladding layers and the complex corrosion atmosphere.

**Figure 6:**
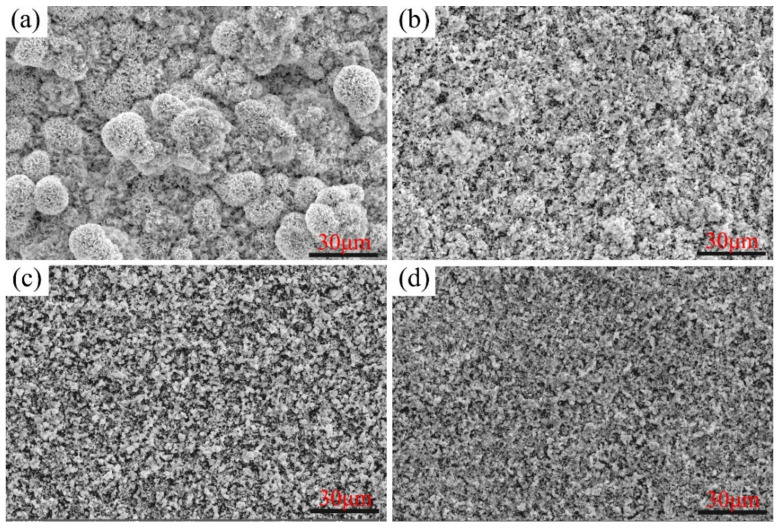
Secondary electron morphology of Ni–xCr–Mo laser-cladding coatings corroded for 168 h at 500 °C: (**a**) C1, (**b**) C2, (**c**) C3, and (**d**) C4.

The surface morphologies of the Ni–xCr–Mo laser-cladding coatings with varying Cr contents corroded at 500 °C for 168 h are depicted in Figure 6, which depicts a loose corrosion product cluster of C1 (Figure 6a) with rough corroded surfaces underneath. According to the linear variation tendency of the high-temperature corrosion kinetics curves presented in Figure 4, the corrosion product cluster of C1 was not sufficiently dense to protect the matrix from further corrosion.

As the Cr content increased to 15 wt.% in C2 (Figure 6b), the corrosion products on the surface were still loose, but the size of corrosion product clusters decreased with the evident reduction in mass gain as compared to those of C1 (Figure 4a). Specifically, this phenomenon was caused by the formation of a dense corrosion layer under the corrosion surface that cannot be observed in Figure 6b. Additionally, the multilayer structure of the corrosion scales is discussed in Section 3.4.

As the Cr content in Ni–xCr–Mo laser-cladding coatings increased up to 20 and 25 wt.%, the corrosion surfaces of C3 and C4 were composed of discontinuous particles that primarily contained Ni and S with an atomic ratio of 1:1 (detailed EDS results are presented in Section 3.4). Upon combining with the XRD patterns presented in Figure 5, the discontinuous particles were determined as NiS particles.

**Figure 7:**
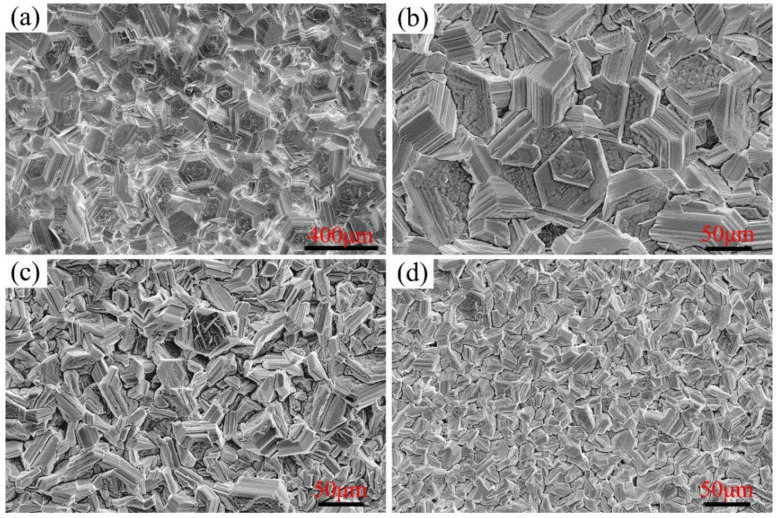
Secondary electron morphology of Ni–xCr–Mo laser-cladding coatings corroded for 168 h at 550 °C: (**a**) C1, (**b**) C2, (**c**) C3, and (**d**) C4.

The surface morphologies of Ni–xCr–Mo laser-cladding layers corroded for 168 h at 550 °C are presented in Figure 7. As compared to Figure 6, the surface morphology varied from the discontinuous particles at 500 °C to a lamellar block corrosion product at 550 °C. Moreover, the size of the corrosion blocks decreased as the Cr content increased in the Ni–xCr–Mo coatings. Furthermore, the EDS results and XRD patterns suggested that the major corrosion products formed on the surface at 550 °C included NiS, similar to those formed at 500 °C.

**Figure 8:**
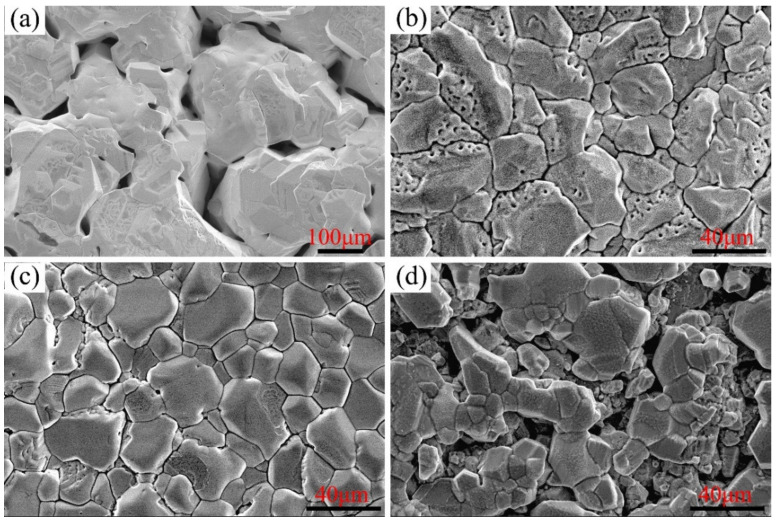
Secondary electron morphology of Ni–xCr–Mo laser-cladding coatings corroded for 168 h at 600 °C: (**a**) C1, (**b**) C2, (**c**) C3, and (**d**) C4.

The surface morphologies of Ni–xCr–Mo laser-cladding layers corroded for 168 h at 600 °C are illustrated in Figure 8, which displays that the surface morphology observed at 600 °C was similar to that detected at 550 °C—composed of block corrosion products. However, the size of the corrosion block at 600 °C was larger than that at 550 °C. Overall, the corrosion surfaces of C1, C2, and C3, as depicted in Figure 8a–c, respectively, were covered by the corrosion products. Nonetheless, the gaps between the corrosion blocks enlarged when the Cr content reached 25 wt.%, as portrayed in Figure 8d.

### 3.4. Cross-Section Morphology after High-Temperature Sulfur Corrosion Test

**Figure 9:**
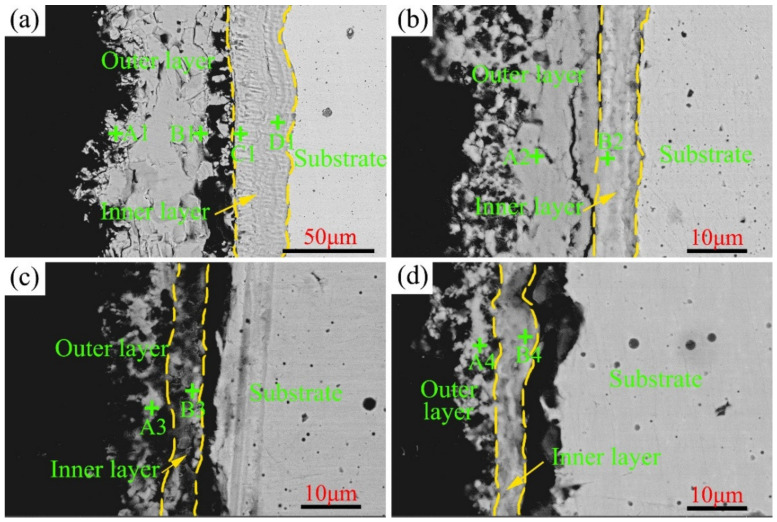
Cross-section morphology of Ni–xCr–Mo laser-cladding coatings under back-scattered mode after the sulfur corrosion test at 500 °C for 168 h: (**a**) C1, (**b**) C2, (**c**) C3, and (**d**) C4.

To better understand the influence of the Cr content on the corrosion resistance of Ni–xCr–Mo laser-cladding coatings, the cross-section morphology after the high-temperature sulfur test at 500–600 °C was observed under the back-scattered mode. As depicted in Figure 9, a two-layer structure, including an outer and inner layer of the corrosion scales, can be observed in C1–C4. The element composition of various sublayers is presented in Table 4. As indicated in Figure 9 and Table 4, the major elements in the outer layer included Ni and S, whereas the predominant elements in the inner layer were Mo, Cr, and O, which shows that Cr is enriched to the metal matrix during the corrosion process, which is consistent with the analysis results of Cr in steel [30,31]. Upon combining with the XRD patterns displayed in Figure 5, the main phase of the outer layer was determined as NiS and that of the inner layer involved the complex oxides of Mo and Cr. The black gap shown in Figure 9c,d between the corrosion scale and substrate was primarily caused by the separation of the corrosion layer during the SEM sample-preparation process.

In general, the element Ni tended to diffuse outward and react with S to form an outer layer of NiS, whereas the elements Mo and Cr tended to enrich in the inner layer and react with O, diffusing inward through the loose NiS outer layer to form an inner scale of complex oxides.

**Figure 10:**
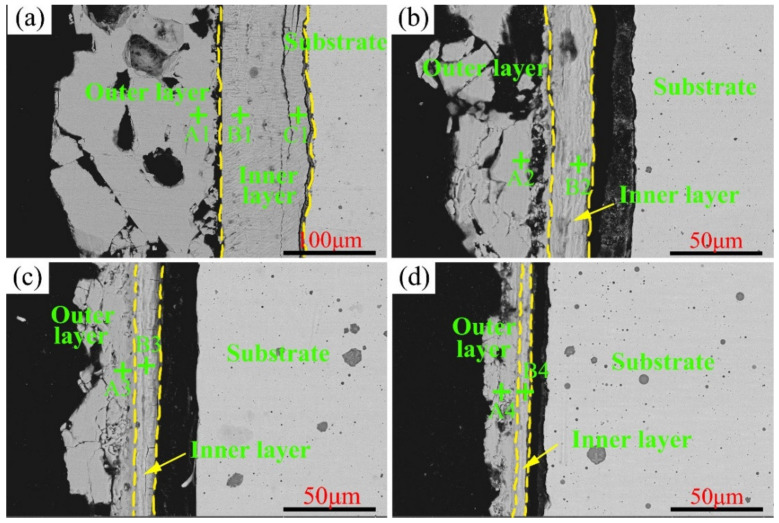
Cross-section morphology of Ni–xCr–Mo laser-cladding coatings observed under back-scattered mode after the sulfur corrosion test at 550 °C for 168 h: (**a**) C1, (**b**) C2, (**c**) C3, and (**d**) C4.

The cross-section morphologies of C1–C4 after the high-temperature sulfur test at 550 °C are illustrated in Figure 10, and the EDS results of the typical points presented in Figure 10 are detailed in Table 5. The two-layered structure observed at 500 °C was detected at 550 °C as well. More specifically, the outer layer was composed of NiS, and the inner layer comprised oxides of Cr and Mo. Notably, the Cr content in the inner layer increased from 15.2 at.% of C1 to 27.1 at.% of C4. The increasing Cr content in the corrosion scales is essential for the corrosion resistance of Ni–xCr–Mo laser-cladding coatings. A higher Cr content reduces the outward diffusion rate of Ni and yields a lower corrosion weight gain, which is consistent with the high-temperature kinetics curves reported in Figure 4b.

**Figure 11:**
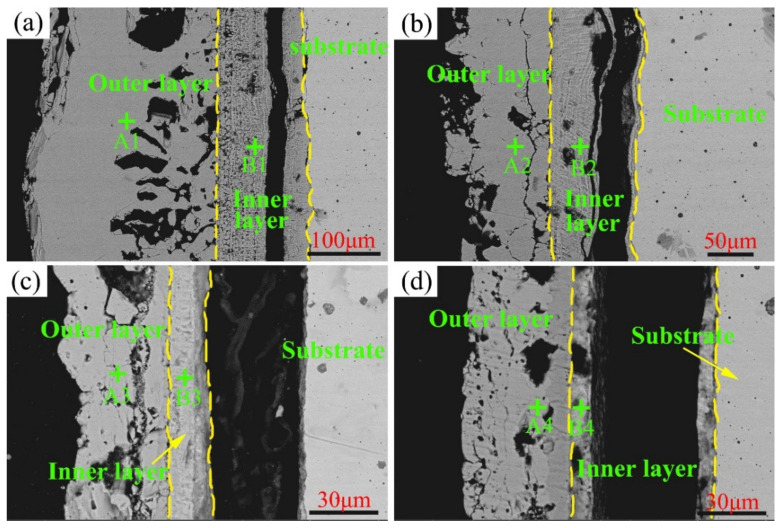
Cross-section morphology of Ni–xCr–Mo laser-cladding coatings observed under back-scattered mode after the sulfur corrosion test at 600 °C for 168 h: (**a**) C1, (**b**) C2, (**c**) C3, and (**d**) C4.

The cross-section morphologies of C1–C4 after high-temperature sulfur tests at 600 °C are depicted in Figure 11, and the EDS results of the corresponding sublayers are detailed in Table 6. As observed, the outer layer comprised sulfides of Ni, and the inner layer contained oxides of Mo and Cr. For the Cr content in the inner layer, the same variation tendency exhibited at 600 °C corresponded to that displayed at 550 °C.

**Figure 12:**
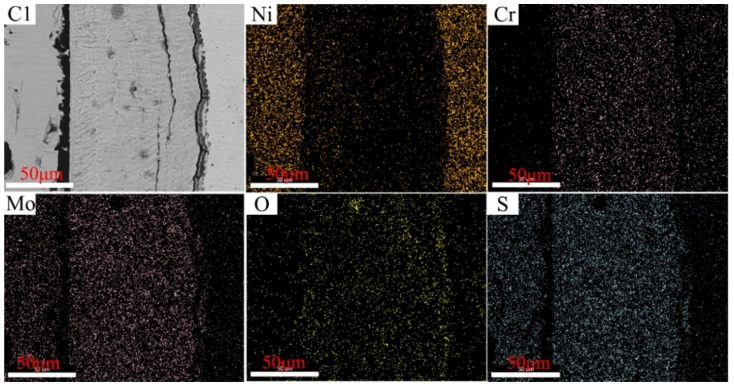
EDS maps for C1 cross-section after the sulfur corrosion test at 550 °C for 168 h.

**Figure 13:**
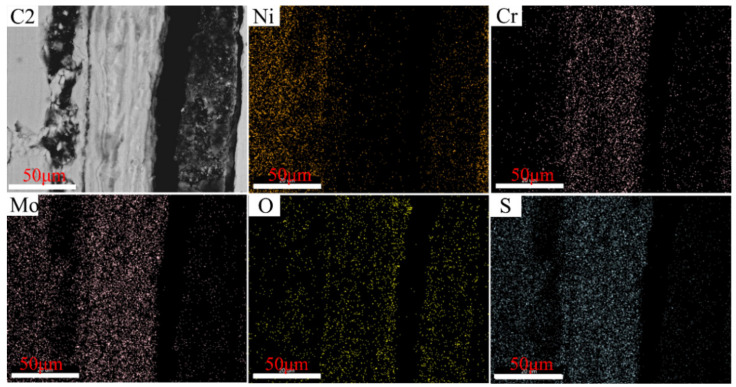
EDS maps of C2 cross-section after the sulfur corrosion test at 550 °C for 168 h.

**Figure 14:**
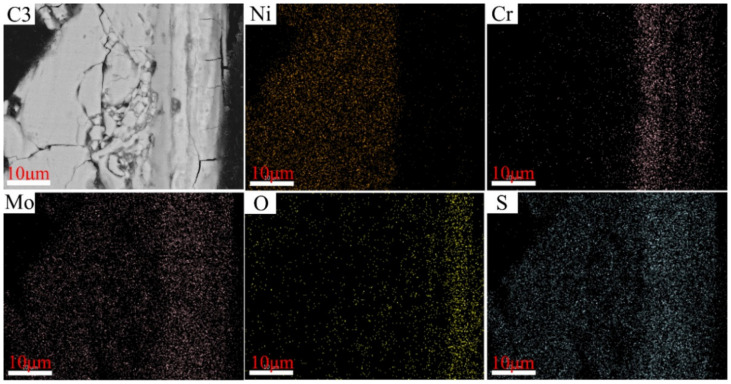
EDS maps for C3 cross-section after the sulfur corrosion test at 550 °C for 168 h.

**Figure 15:**
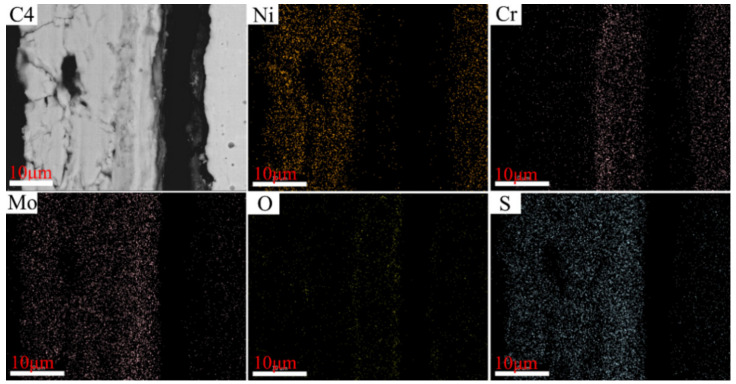
EDS maps of C4 cross-section after the sulfur corrosion test at 550 °C for 168 h.

The distributions of the elements in the cross-section of C1–C4 after corrosion tests conducted at 550 °C are illustrated in Figure 12, Figure 13, Figure 14 and Figure 15, wherein the corrosion layer is presented on the left-hand side and the substrate is depicted on the right-hand side. As discussed above, the element distributions after corrosion at 550 and 600 °C were similar to that at 550 °C. Thus, only that at 550 °C is illustrated here to avoid repetition.

As depicted in Figure 12, Figure 13, Figure 14 and Figure 15, the element Ni enriched the outer layer, whereas the element Cr enriched the inner layer. More importantly, the Cr content in the inner layer increased with that of the Ni–xCr–Mo laser-cladding coatings, which was consistent with the EDS atomic fraction listed in Table 5. As shown in Figure 15, the Ni content in the inner layer of C4 was slightly lower than that of C1 (Figure 12), which was also confirmed in Table 5. This phenomenon indicated that a higher Cr content, for example C4, in the Ni–xCr–Mo laser-cladding coatings resulted in a denser inner layer containing Cr oxides, and the denser inner layer could prevent the Ni^2+^ from diffusing outward to react with the S^2−^ and result in a lower corrosion mass gain (Figure 4). Consequently, the corrosion resistance of Ni–xCr–Mo laser-cladding coatings increased with the Cr content.

## 4. Discussion

### 4.1. Effect of Cr Content on Corrosion Resistance of Ni–xCr–Mo Laser-Cladding Coatings

To explore the effect of Cr content on the corrosion resistance of Ni–xCr–Mo laser-cladding coatings, high-temperature sulfur corrosion accelerated tests were conducted at 500–600 °C of Ni–xCr–Mo laser-cladding coatings (x = 10, 15, 20 and 25 wt.%) using an atmosphere-controlled furnace simulating a low-nitrogen combustion environment. During acceleration experiments, the corrosion gas mixture was continuously provided to supply reactants of the sulfide and oxidation reactions.

The mass gain of Ni–xCr–Mo laser-cladding coatings versus exposure period at 500–600 °C is plotted in Figure 4, which revealed that the corrosion at 600 °C was more severe than that at 500 and 550 °C. As the test period (168 h) was much shorter than the service period (several years) of the water wall of boilers, an extrapolation based on fitting available from the experimental data was required to obtain a reasonable trend of corrosion growth with the exposure period. The experimental weight-gain data can be fitted using the following Equation [32]:

*Y* = A*t*^n^ (2)

where *Y* denotes the weight variation of the laser-cladding coating in mg/cm^2^, A represents the rate constant in mg/(cm^2^·h), *t* is the exposure period in h, and n denotes a time exponent describing the time dependence of the corrosion growth. Both A and n are detailed in Table 7. For *n* = 1, the fitting curves depict a linear law indicating the loose and porous corrosion scales that cannot prevent further corrosion of the substrate. Overall, the corrosion was controlled by a chemical reaction, and the corrosion rate was equal to the chemical reaction rate. For *n* = 0.5, the fitting curves followed the parabolic law, indicating the formation of a dense corrosion layer that can decrease the corrosion rate and protect the matrix. The corrosion process was controlled by the diffusion rate of the corrosion gas and metal atoms via the corrosion layers on the surface [32,33,34]. As indicated in Table 7, the weight gain of C1 follows the linear law at 500–600 °C, which signifies that 10 wt.% Cr did not positively influence the corrosion resistance. However, samples C2–C4 displayed near-parabolic law at 500–600 °C because the time exponent was in the range of 0.499–0.697, which is close to 0.5.

It should be noted that the content density of Cr in the inner layer increased with the increasing Cr content, while the content density of Ni in the inner layer decreased with the increasing Cr content from 10 to 25 wt.%. At 550 °C, according to the EDS spectra of C1–C4 in Table 5, the Cr content in the dense inner corrosion layer of C1–C4 increased from 15.2, 19.3, and 25 at.% to 27.1 at.%, whereas the Ni content in the inner corrosion layer diminished from 4.7 to 1.6 at.%. At 600 °C, the Cr content in the inner layer increased from 12.7 to 35.5 at.% as the Cr content increased from 10 to 25 wt.%. For a Cr content of 10 wt.%, Ni content in the inner corrosion layer could increase by more than 10% (as stated in Table 6), and the EDS maps displayed in Figure 12 indicated a high Ni content in the inner corrosion layer of the C1 cross-section. In contrast, for Cr content ≥ 15 wt.%, the Ni content in the inner corrosion layer remarkably decreased to 2–3.6 at.%. The content density of Cr and Ni in the inner layer for C1 and C2 corroded at 500 °C showed the same variation tendency as that at 550 and 600 °C, while the content density of Cr and Ni in the inner layer for C3 and C4 corroded at 500 °C did not display the same tendency. This is because the corrosion scale is too thin, and the EDS results are affected by the matrix.

Overall, at the same corrosion temperature, for instance 550 °C, the Ni content in the inner layer of C1–C4 decreased from 4.8 to 1.6 at.% as the Cr content increased from 10 to 25 wt.% (as shown in Table 5). The time exponent of C1 was 1 while that of C2–C4 tended to be 0.5, and the rate constant of C2–C4 decreased from 0.7541 to 0.289 as the Cr content increased from 10 to 25 wt.%. This result confirmed that a higher Cr content results in the formation of a denser corrosion layer that can prevent the mutual diffusion of corrosive gas and Ni atoms, which consequently yields a lower corrosion rate.

**Figure 16:**
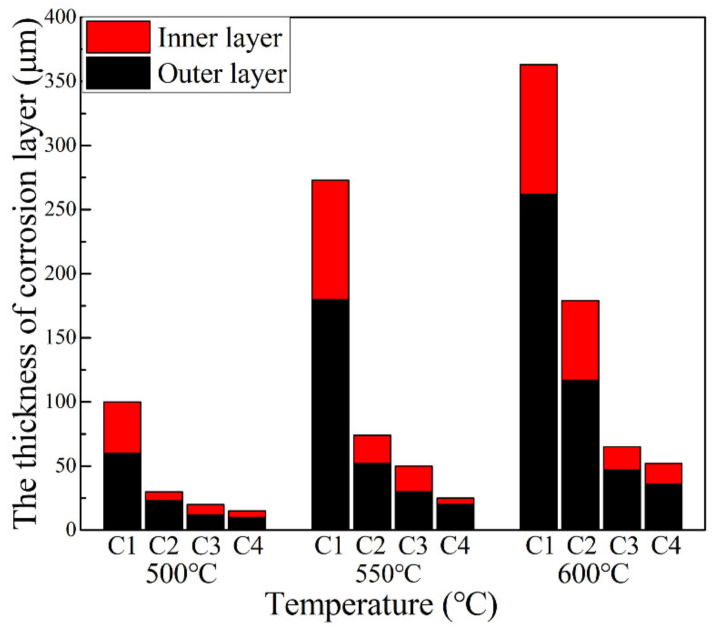
Bar diagram of thickness variation of outer, inner, and total corrosion layers.

The thickness variation of the outer, inner, and total corrosion layers of Ni–xCr–Mo laser-cladding coatings after conducting the corrosion tests at 500–600 °C is exhibited in Figure 16, which depicts that the total thickness of the corrosion scales increased with the corrosion temperature. For the same corrosion temperature, the thickness of the outer and inner layers decreased with the increasing Cr content. As Cr content increased from 10 to 25 wt.%, the thickness of the inner and outer layers prominently decreased, which implied a significant improvement in the corrosion resistance of the material. This phenomenon is consistent with the kinetic curve fitting law reported in Table 7 and is evidence that a higher Cr content results in a lower corrosion rate.

### 4.2. Corrosion Behavior of Ni–xCr–Mo Laser-Cladding Coatings

During the high-temperature corrosion test, S is generated by the reaction between H_2_S and O_2_, as shown in the chemical reaction in Equation (3) [35]. The generated S reacts with Ni and Cr to form the sulfides of Ni and Cr, as detected by XRD patterns in Figure 5. The possible reactions are shown in chemical reactions in Equations (4)–(6). In a previous study [36,37], Cr and Mo were more likely to react with O_2_ to form the oxides of Cr and Mo, as shown in chemical reactions in Equations (7)–(9). However, in this study, the oxides of Cr and Mo could not be detected in Figure 5 because the outer layer of the corrosion scales was too thick, so the X-ray could not penetrate the surface to detect the underlying oxidation products. The EDS results in Table 4, Table 5 and Table 6 show the enrichment of Cr and Mo in the inner layer, which reflects the selective corrosion between Cr/Mo and O. The formation of NiMo_2_S_4_ and CrMo_2_S_4_ is due to the interactions between various elements and single corrosion products [36].

2H_2_S + O_2_ = 2S + 2H_2_O (3)

Ni + S = NiS(4)

2Cr + 5S = Cr_2_S_5_ (5)

5Cr + 8S = Cr_5_S_8_ (6)

Mo + O_2_ = MoO_2_ (7)

2Mo + 3O_2_ = 2MoO_3_ (8)

4Cr + 3O_2_ = Cr_2_O_3_ (9)

**Figure 17:**
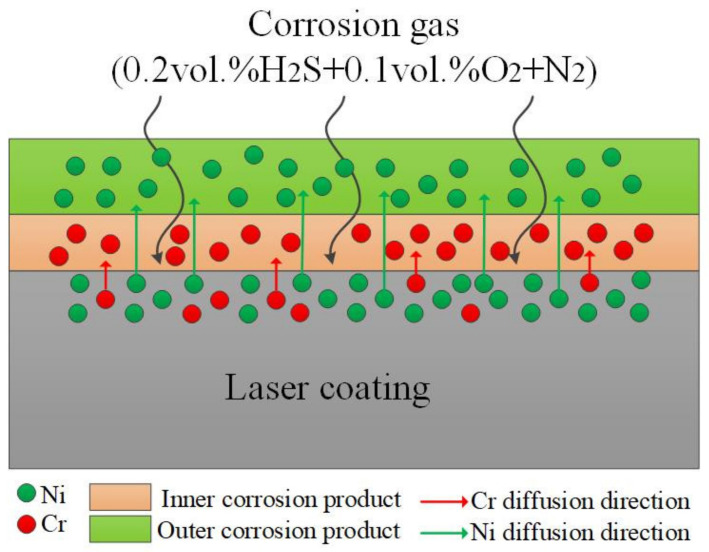
Corrosion principle, diagram of the cladding layer.

During the corrosion process, the Cr element gradually accumulated in the inner layer and formed the dense Cr-based corrosion products on the matrix surface. Moreover, the diffusion direction of the Ni^2+^ is from inward to outward, passing through the dense corrosion product layer and forming a loose Ni-sulfide corrosion layer above the dense corrosion product layer, as graphically illustrated in Figure 17. Therefore, the diffusion rate of Ni^2+^ in the dense corrosion layer is a vital aspect influencing the corrosion rate. Based on the above discussion, the linear corrosion curve for a Cr content of 10 wt.% implies that the corrosion layer formed on the matrix surface during the corrosion process cannot curtail the diffusion of the Ni^2+^, which can still effortlessly pass through the inner corrosion layer to contact the external corrosion gas and generate vulcanized objects. This finding revealed that the laser coating did not deliver appropriate corrosion performance for a Cr content of 10 wt.%.

In a case where the Cr content in the laser-cladding layer increased to more than 15 wt.%, the corrosion rate of the cladding layer decreased significantly in comparison to that of C1 (Figure 4), and the mass-gain curve was parabolic. Additionally, the Cr element in the inner corrosion layer started to increase, and the amount of Ni in the inner corrosion layer was ~4.1 at.%, thus suggesting that the Cr-enriched corrosion products in the inner layer could effectively restrict the diffusion of Ni^2+^, and the Ni^2+^ cannot conveniently pass through the inner corrosion layer. This phenomenon indicated that a greater Cr content in the Ni–xCr–Mo laser-cladding coatings produces a denser inner layer containing oxides of Cr, and this is the fundamental reason for the improvement in the corrosion resistance of the cladding layer. Comparatively, the diffusion of Ni^2+^ becomes challenging as the Cr content in the inner corrosion products increases. Therefore, the corrosion resistance of the cladding layer is improved (as the content of Mo element remains constant in each cladding layer, and the current study mainly focuses on characterizing the influence of the Cr element on corrosion resistance, the impact of Mo in this aspect was not discussed in this paper).

In general, the corrosion products of Ni–xCr–Mo laser coatings are primarily divided into two layers: the inner layer of dense corrosion product and the outer layer of loose corrosion product. More specifically, the outer layer is composed of Ni sulfides with low Cr content, whereas the inner layer mainly contains oxides of Cr and Mo with low Ni content. The corrosion resistance of Ni–xCr–Mo laser-cladding coatings decreased with the increasing corrosion temperature. Conversely, the corrosion resistance improved with the increasing Cr content for the same corrosion temperature. When the Cr content reached 15 wt.%, the corrosion resistance displayed an evident improvement, and the kinetic curves tended to exhibit a parabolic law. Overall, the 15 wt.% Cr was considered the critical minimum value to obtain adequate corrosion resistance and the 30 wt.% Cr was considered the limited maximum content for improved morphology without large prominent pores.

## 5. Conclusions

The sulfur corrosion tests of Ni–xCr–Mo laser-cladding coatings were conducted in an H_2_S reducing atmosphere at 500–600 °C within the simulated atmosphere furnace. The phase construction and microstructure evolution of the corrosion products were analyzed to characterize the influence of Cr content on the corrosion resistance. The major conclusions of this study are summarized as follows:

(1) Ni–xCr–Mo laser-cladding coatings were composed of (Ni, Cr, Mo)ss solid solution. The Mo-rich network phase became coarser and the number of pores increased with the Cr content. The experimental results indicated that 25 wt.% Cr is the limited maximum content to obtain a suitable morphology without large prominent pores in terms of the fabrication process.
(2) The corrosion resistance of Ni–xCr–Mo laser-cladding coatings increased with the Cr content, and 15 wt.% Cr corresponded to the critical minimum content for parabolic corrosion law and adequate corrosion resistance ability.
(3) A two-layer structure of the corrosion scales was observed because of the selective corrosion phenomenon between Ni and S and Mo/Cr and O. The outer layer contained loose NiS, whereas the inner layer contained dense oxides of Cr and Mo.

## Figures and Tables

**Table 1:** Chemical composition of laser-cladding Ni-based powder (wt.%).

Samples	Ni	Cr	Mo	Si
C1	73	10	16	1
C2	68	15	16	1
C3	63	20	16	1
C4	58	25	16	1
C5	53	30	16	1

**Table 2:** Element content on the surface of Ni–xCr–Mo coatings in mass fraction (wt.%).

Samples	Ni	Cr	Mo
C1	77.5	9.5	13
C2	73.1	14	12.9
C3	67	19	14
C4	60.9	24.5	14.6

**Table 3:** Element content for typical phases of Ni–xCr–Mo coatings displayed in Figure 1.

Samples	Spot	Ni (at.%)	Cr (at.%)	Mo (at.%)
C1	A1	82.2	10.2	7.6
	B1	79.4	10.8	9.8
C2	A2	77.7	14.9	7.4
	B2	74.2	16.1	9.8
C3	A3	68.1	22.9	9
	B3	65.4	23.6	11
C4	A4	64.3	26.9	8.9
	B4	60.7	28	11.2

**Table 4:** EDS results (at.%) of typical points displayed in Figure 9.

Samples	Points	Ni	Cr	Mo	O	S
C1	A1	42.1	1.7	13.8	0	42.4
	B1	38.8	3.2	16.6	0.8	40.7
	C1	10.2	14.8	19.9	31.4	23.7
	D1	13.9	15.1	16.7	27.3	27.1
C2	A2	57.6	2.2	20	0.8	19.5
	B2	4	19.9	31.5	9.9	34.6
C3	A3	54.9	3.3	19.5	10.5	11.8
	B3	4.1	16.8	27.6	28.6	23
C4	A4	49.7	1.5	11.8	6.5	30.4
	B4	4	14.1	23.4	40.1	18.4

**Table 5:** EDS results (at.%) of typical points displayed in Figure 10.

Samples	Points	Ni	Cr	Mo	O	S
C1	A1	52.2	0.8	9.7	4.7	32.2
	B1	4.7	15.2	15.7	42.4	22
	C1	4.8	15.8	16.2	42.2	16.2
C2	A2	54.8	1.4	11.7	0.5	31.6
	B2	2.6	19.3	20	31.3	22.5
C3	A3	40.1	1.5	13.3	0.1	45.1
	B3	1.8	25	16.2	22.9	34.1
C4	A4	37.4	4.8	10.5	45.9	45.9
	B4	1.6	27.1	17.9	23	30.4

**Table 6:** EDS results (at.%) of typical points displayed in Figure 11.

Samples	Points	Ni	Cr	Mo	O	S
C1	A1	44.9	1	8.4	0	45.7
	B1	11	12.7	13	44.2	19.1
C2	A2	55.8	1.1	13.7	0.2	29.2
	B2	3.6	21.3	15.8	42.9	16.4
C3	A3	41.6	1.3	8.3	0	48.8
	B3	2.3	23	15.6	31.6	27.5
C4	A4	49	4.1	9.4	0.5	37
	B4	2	35.5	16.7	28.1	17.7

**Table 7:** Fitting curve parameters of high-temperature kinetics curves plotted in Figure 4.

Sample	500 °C	550 °C	600 °C
C1	*Y* = 0.786 + 0.088*t* R^2^ = 0.992	*Y* = 1.875 + 0.244*t* R^2^ = 0.9995	*Y* = 2.958 + 0.393*t* R^2^ = 0.993
C2	*Y* = 0.133*t*^0.631^ R^2^ = 0.985	*Y* = 0.7541*t*^0.513^ R^2^ = 0.988	*Y* = 0.469*t*^0.697^ R^2^ = 0.994
C3	*Y* = 0.121*t*^0.541^ R^2^ = 0.984	*Y* = 0.349*t*^0.507^ R^2^ = 0.994	*Y* = 0.440*t*^0.624^ R^2^ = 0.994
C4	*Y* = 0.077*t*^0.532^ R^2^ = 0.998	*Y* = 0.289*t*^0.499^ R^2^ = 0.997	*Y* = 0.351*t*^0.511^ R^2^ = 0.986

## Data Availability

Not applicable.
